# The Climate Change, Food Security and Human Health Nexus in Canada: A Framework to Protect Population Health

**DOI:** 10.3390/ijerph16142531

**Published:** 2019-07-16

**Authors:** Rebekka Schnitter, Peter Berry

**Affiliations:** 1 Climate Change and Innovation Bureau, Health Canada, 269 Laurier Ave. W, Ottawa, ON K1A 0P8, Canada; 2 Department of Geography and Environmental Management, University of Waterloo, 200 University, Avenue W, Waterloo, ON N2L 3G1, Canada

**Keywords:** food security, food systems, food insecurity, health, climate change, framework

## Abstract

Climate change impacts on the Canadian food system pose risks to human health. Little attention has been paid to the climate change, food security, and human health nexus, resulting in a number of knowledge gaps regarding food system components that are most vulnerable to climate change. The lack of understanding of key dynamics and possible future impacts challenges the ability of public health officials and partners in other sectors to prepare Canadians for future health risks. A series of literature reviews were conducted to establish the relationship between climate change, food security, and human health, and to identify vulnerabilities within the Canadian food system. Evidence suggests that key activities within the food system are vulnerable to climate change. The pathways in which climate change impacts travel through the food system and affect the critical dimensions of food security to influence human health outcomes are complex. Climate-related disruptions in the food system can indirectly impact human health by diminishing food security, which is a key determinant of health. Human health may also be directly affected by the physical effects of climate change on the food system, primarily related to the impacts on nutrition and foodborne illnesses. In this study, we propose a novel analytical framework to study and respond to the climate change, food security, and human health nexus. This work is intended to help public health officials, researchers, and relevant stakeholders investigate and understand current and future risks, and inform adaptation efforts to protect the health of Canadians.

## 1. Introduction

It is well established that slow- and rapid-onset climate change effects will cause adverse impacts on natural and human systems. Health risks associated with extreme weather and climate events have already been observed, and are increasing globally and in Canada. For example, in 2003, a heat wave in Europe resulted in over 70,000 deaths [1]; flooding in High River, Alberta, in 2013 resulted in a number of adverse mental health outcomes [2]; and Lyme disease cases increased by over 1300% from 2009 to 2017 in Canada [3]. The impacts of climate change will vary across the country, as will demographic, socio-economic, infrastructural, institutional capacity, and health system resiliency. Thus, there will be regional variation in terms of the strength and severity of associated health impacts. Indirect and direct effects of climate change such as temperature extremes, extreme weather events, air pollution, infectious diseases transmitted by vectors, stratospheric ozone depletion, and contamination of food and water [4,5] are associated with health risks that are of significant concern in Canada.

The impacts of a changing climate will have direct and indirect effects on global and domestic food systems [6,7]. Recent scientific evidence has led to increased concern about the possible impacts of climate change on food security. In its fifth assessment report, the Intergovernmental Panel on Climate Change (IPCC) indicated that climate change would have significant effects on food in the near-to-medium term [8]. On a global level, it has been suggested that climate change impacts on undernutrition constitute this century’s greatest threat to health [9]. An examination of 30 of the most vulnerable countries to climate change impacts on undernutrition found that the number of undernourished people increased by approximately 6%, from 398 million people in 1990 to 422 million people in 2016 [9]. The World Health Organization (WHO) estimates that without adaptation, climate change will result in 7.5 million more cases of stunted children by 2030 and 10.1 million by 2050 [10]. Furthermore, an additional 4.8 million cases of undernutrition in children under 5 years of age is projected by 2050 due to climate change [11]. Currently, world hunger is on the rise and climate-related shocks have exacerbated this trend [12]. Extreme weather events were a major driver of the drastic increase in the number of people facing crisis level food insecurity in 2015 (~80 million people) and 2016 (~108 million people) [13]. 

Evidence suggests that climate change is impacting food security in Canada, particularly in northern and Indigenous communities [4,14]. Observed climate impacts related to reduced duration and thickness of sea and lake ice, thawing permafrost, more unpredictable weather, freezing rain and wildfires, shorter winter seasons, and hotter summers can threaten food safety and security, for example, by challenging hunting and gathering activities and traditional food storage practices [14,15]. Climate change impacts are also contributing to a change in the abundance and geographic distribution of populations of traditionally harvested species, such as caribou [4,14]. In Manitoba, many of the province’s 25 northern communities have experienced supply shortages of healthy food products due to warming conditions resulting in deteriorating ice roads [16]. 

The pathways in which climate change impacts travel through the food system and affect the critical dimensions of food security to influence human health outcomes are complex. Some pathways are understood well, while others have many knowledge gaps. Climate-related disruptions in the food system can indirectly impact human health by diminishing food security, which is a key determinant of health. Human health may also be directly affected by the physical effects of climate change on the food system, primarily through pathways of food safety and nutrition.

In Canada, little attention has been paid to this relationship in climate change research, resulting in a number of knowledge gaps regarding components of the food system most vulnerable to climate change, current and future impacts in Canada from warming of the climate [4], and the most effective strategies for adapting. This lack of understanding of the key dynamics and possible future impacts challenges the ability of public health officials and partners in other sectors to prepare Canadians for future risks to health. 

The relationship between climate change, the food system, food security, and human health is dynamic and multi-sectoral. This creates conceptualization difficulties in recognizing vulnerabilities. The factors leading to health outcomes interact in complex systems that need to be understood. Challenges therefore exist in describing, measuring, and projecting future health risks associated with climate change impacts on food security [17]. 

To address these challenges, this paper presents a novel analytical framework to study and respond to the climate change, food security, and human health nexus. Current levels of food insecurity in Canada are presented, along with evidence of impacts on health and wellbeing. The framework is then presented, highlighting the complex and multi-sectoral relationships between climate change, food security, and human health. Examples of the Canadian food system’s components that may be vulnerable to disruption by climate change impacts are identified, followed by analysis of how key pathways of vulnerability can influence food security and human health. 

A number of frameworks exist that demonstrate the relationships between climate change and food security or food systems [6,15,18,19], and to some extent, health or nutrition [20,21,22]. However, to the best of our knowledge, there is no existing framework that conceptualizes the nexus of climate change, food security, and human health in Canada. The proposed framework is intended to help public health officials, researchers, and relevant stakeholders investigate and understand current and future risks to food security from climate change, to inform efforts to prepare Canadians and their communities for possible health impacts.

## 2. Background: Food Security in Canada 

In 1996, Canada adopted the Rome Declaration on World Food Security and the World Food Summit Plan of Action. The declaration indicates that food security exists when “…all people, at all times, have physical, social and economic access to sufficient, safe and nutritious food which meets their dietary needs and food preferences for an active and healthy life” [23]. Three primary dimensions must be met in order for food security to exist: availability, accessibility, and utilization of food [24]. 

*Food availability* concerns the “physical presence of food” [25] and includes the quantity, quality, and type of food available to individuals. Food production, food distribution, and the exchange of food are key elements of food availability [18,19,24,26]. The ability of individuals to acquire the quantity, quality, and type of food necessary for an active and healthy life is captured in the dimension of *food accessibility*. Key elements of food accessibility include affordability, allocation, and preference [19,24,25]. *Food utilization* refers to the ability to effectively use, consume, and benefit from food. Food safety as well as the nutritional and social value of food, are considered to be key elements of food utilization [18,19,24]. 

The temporal stability of the three dimensions described above is also required to achieve food security [18]. Indeed, food insecurity refers to “both the inability to secure an adequate diet today and the risk of being unable to do so in the future” [14], indicating the importance of the long-term stability of food availability, accessibility, and utilization. *Food insecurity* exists when any of the primary dimensions are unmet.

**Figure 1:**
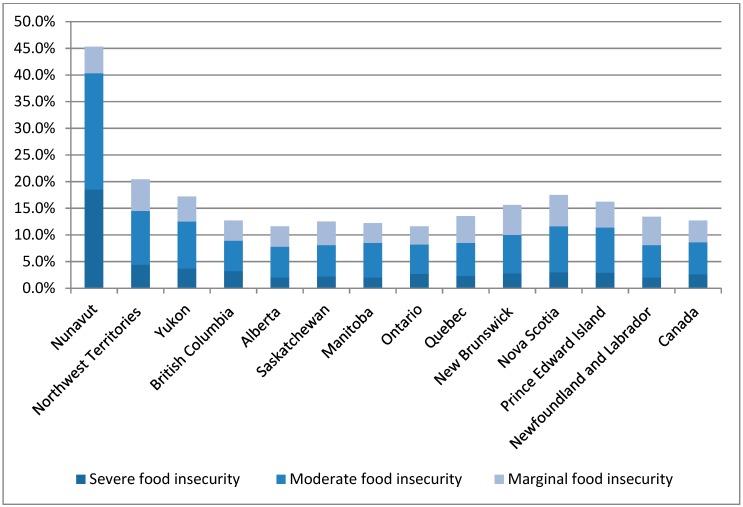
Percentage of households with food insecurity, by province or territory, Canada 2011–2012 [27].

In Canada, approximately 12.6% of households were considered to be food insecure in 2011–2012, representing 2.8 million adults and 1.5 million children under the age of 18 [27]. These rates are an underestimation given that people living on First Nations reserves, full time members of the Canadian Forces, individuals in prisons or care facilities, and the homeless population are not included in this data [27,28]. As illustrated in Figure 1, rates of food insecurity are significantly higher in the Canadian North.

There is much evidence that suggests linkages and associations between food security and health conditions. For example, issues related to birth outcomes and maternal health, child development, chronic diseases, mental health, and emotional wellbeing, and increases in health care costs may emerge in food insecure households (Table 1) [29]. 

A nutritious diet, a key aspect of food security [30], plays a significant role in an individual’s health and wellbeing. In Canada, an unhealthy diet is considered a leading risk for death and disability [31] and is associated with an economic burden of approximately 13.8 billion dollars a year in Canada [32]. Increased consumption of processed foods and beverages that contribute to excess intake of sodium, sugars, and saturated fats and insufficient intakes of whole grains, nuts and seeds, fruits, and vegetables are key drivers of unhealthy diets [31]. The Global Burden of Disease Study reported that in 2016, dietary risk factors resulted in more than 800,000 years of disability and the death of approximately 48,000 Canadians [33,34].

## 3. Methods 

An analytical framework was developed to illustrate the complex relationship between climate change impacts and the food system, and how this relationship influences food security and human health in Canada. In order to appropriately capture the breadth of the climate change, food security, human health nexus, and the nuances of the relationship, multiple literature searches were conducted. The databases Medline and Embase were searched via Ovid between 2007 and September 2017. To understand the possible impacts of climate change on the Canadian food system and the subsequent implications for human health, four search strategies were developed in consultation with a research librarian at Health Canada’s Health Library: (1) climate change + food security + Canada, (2) climate change + food security + health, (3) food security + health + Canada, and (4) food systems + climate change + food security.

An additional literature search was conducted between 2000 and April 2019 to identify frameworks that conceptualize the relationship between climate change, food security, and human health. Embase and Medline were searched via Ovid. Another simplified keyword search was conducted in AGRICOLA, BIOSIS Previews, Food Science and Technology Abstracts, Global Health, PsycINFO, and Social Policy and Practice. 

## 4. Results

### 4.1. The Climate Change, Food Security, and Human Health Nexus Framework

Few studies and frameworks exist to increase our understanding of the possible impacts of climate change on food security and human health. Commonly, researchers examine the impacts of climate change on food systems or food security, but rarely are implications for human health included in the analysis. Thus, there is no comprehensive or common approach to studying the climate change, food security, and human health nexus, resulting in important knowledge gaps that hinder effective adaptation efforts.

**Figure 2:**
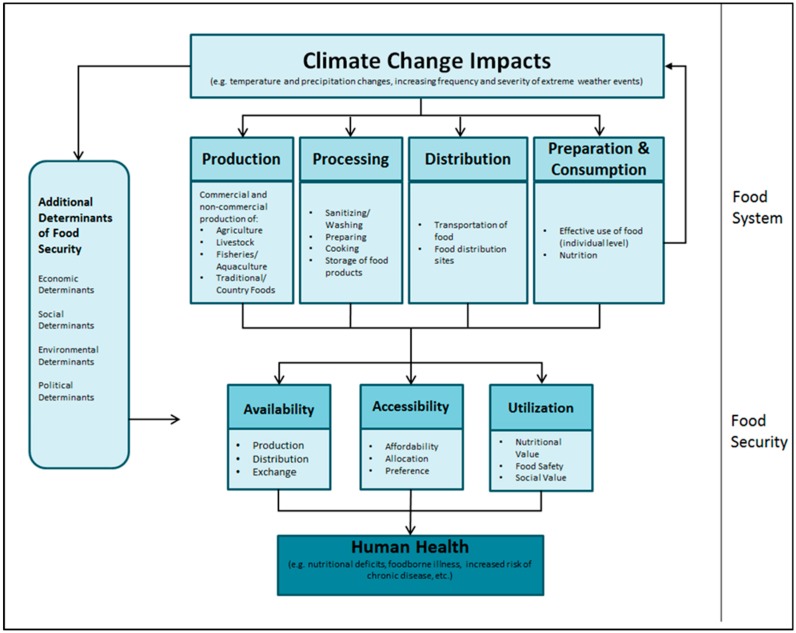
Food security, climate change, and human health nexus framework.

The linkages and drivers between climate change, food security, and human health are presented in a new framework (Figure 2). The framework illustrates the nexus from a food system perspective, highlighting the direct and indirect pathways in which the physical impacts of climate change may work through the food system to influence food security and, subsequently, human health. By identifying the different sectors of the food system, the framework demonstrates that a multi-sectoral approach is necessary for effective adaptation and resiliency building in response to climate change.

The following text provides an explanation of the main components of the framework. Table 2 summarizes potential vulnerabilities to key food system components from climate change impacts. Given the limited literature available in a Canadian context, examples from international studies are used where relevant. Evidence indicates that every component of the food system—production, processing, distribution, preparation and consumption—is climate sensitive and can experience impacts from climate change effects. These impacts can cause disruption and challenges to critical activities of the food system. 

### 4.2. Food Production 

Food production is an essential component of the food system, it encompasses commercial and non-commercial agriculture, livestock, fisheries and aquaculture sectors, as well as the hunting, fishing and harvesting of traditional Indigenous foods. The food production sector is the starting point of the food system, providing the raw ingredients for processing and consumption. Ultimately, if the productivity of the food production sector declines, the availability and supply of food may be challenged. This would have impacts on food security status and, subsequently, human health. 

Climate conditions such as rainfall and temperature have a primary influence on food production through impacts on water yields and flows, which are often modulated by irrigation and other water management techniques [17]. Other climate-related factors that can affect food production, and hence food security, include impacts on freshwater, biodiversity, soil degradation, fisheries, and carbon dioxide fertilization, with attendant impacts on nutritional food quality [35]. Some research suggests that climate change may create opportunities for Canadian food production [8,36]. For example, some regions in Canada may experience an increased growing season and lengthened outdoor feeding season for livestock, which could positively affect food production [36,37]. However, climate projections suggest an increased risk of drought in some regions of Canada, including the Prairies and British Columbia. In addition, future impacts from invasive pests and diseases, and transportation disruptions could put pressures on Canadian food production [38]. On the global level, any positive effects resulting from a warming climate are expected to be significantly outweighed by their adverse effects on the food system [39]. 

### 4.3. Food Processing

Food processing involves the transformation of raw food inputs into food products that are used in the preparation of meals or directly consumed by individuals. The food processing sector has a direct link to human health as its operations include washing, sanitizing, and preparing food that is safe for human consumption. Limited research has been conducted on climate change impacts to the food processing sector, both globally and in the Canadian context. Existing evidence suggests that climate change impacts may cause disruption to the stable supply of necessary resources, and inputs for processing operations and extreme weather events can cause physical damage to processing facilities [7,38,40,41]. This sector is an essential component of food security, supporting the key dimensions of availability, accessibility, and utilization.

### 4.4. Food Distribution

The distribution of food is a critical component of food security as it links food products to consumers, directly supporting the availability and accessibility dimensions of food security. Climate change can disrupt food distribution networks through acute shocks such as extreme weather events, as well as through creeping climatic changes [42]. A national climate change assessment conducted on Canada’s transportation sector in 2016 concluded that climate change will affect all modes of transportation, across the country [42]. Challenges to transportation infrastructure can undermine food security, and regions that are rural or remote—with a low capacity to produce and/or store food products locally—may be particularly vulnerable. Urban transportation infrastructure and public transportation systems, which are relied upon by many Canadians to access food distribution sites, such as grocery stores and markets, are also vulnerable to climate change [42]. Individuals that reside in food deserts—neighborhoods that are characterized by low income households and are underserved in terms of food distribution sites and public transportation infrastructure [43]—are particularly impacted by such disruptions.

### 4.5. Food Preparation and Consumption 

The food preparation and consumption component of the food system directly supports the utilization dimension of food security. Food utilization refers to the need for “available and accessible food to fulfill the cultural, religious, health and nutrition needs of the population” [43]. This suggests that food needs to be safe to consume, be prepared in a culturally appropriate manner, while also providing adequate nutritional value in order for food security to exist. 

Food safety and nutrition may be the pathways through which climate change impacts have the most direct effect on human health [20]. Nutrition may be challenged as climate change can contribute to shifts in diet composition and diversity [14,44]. For example, country and traditional foods provide important nutritional value and contribute to healthy diets for Indigenous populations [14,45]. In northern Indigenous communities in Canada, there has been an observed diet shift from country and traditional foods, to market foods. Climate change impacts on the availability and quality of traditional foods may contribute to this shift [14]. Market foods are often prepared, processed, and/or frozen, while fresh foods such as fruits and vegetables are not always available [14,46]. Foods purchased and consumed are often less nutritious and high in sodium, sugars, and fat, which may contribute to various forms of malnutrition. 

As the framework suggests (Figure 2), proper functioning of the food system is integral for food security by supporting the critical dimensions of availability, accessibility, and utilization of food [24,26,58]. Given this relationship, food security may become compromised should any component of the food system experience disruption or stress in such a way that limits the accessibility, availability, and or proper utilization of food [26,46,58]. Ericksen [19] identifies three primary elements for each of the dimensions of food security, demonstrating the relationship with the food system. The primary elements also have relevance for human health outcomes (Table 3). 

Canada is part of a global food system, exporting and importing raw and prepared food products from other regions of the world [59]. Although the severity or magnitude of some risks caused by climate change to Canada’s food system may be lower compared to other countries, the nature of the global food system suggests that climate change impacts and risks in other regions can have implications for Canadian food security, including food supply and food safety [6]. 

The framework highlights that the presence and strength of food security is influenced by a number of different factors, including social, political, environmental, and economic determinants [19]. Evidence suggests, however, that climate change is increasingly becoming a significant stressor and influence on food security, through its impact on the food system [58]. Furthermore, climate change is a threat multiplier and can have impacts on the other factors that contribute to and influence food security status. 

The framework also acknowledges that the food system is a major source of greenhouse gas emissions, and thus, an important driver of climate change [30,39]. It is estimated that a quarter of global emissions are from food system operations and activities [60]. Furthermore, food systems are resource intensive, requiring significant water and energy inputs to sustain operations and activities. Industrial food systems can also contribute to unsustainable land use and deforestation [30]. 

Traditional and country foods have long been established as an important “enabler of food security and health” [14] for Indigenous communities and provide both spiritual and nutritional value [14,61]. There are unique food security considerations that have not traditionally been included in conceptualizations of western food systems and food security concepts [62]. While this framework does include traditional and country foods and related activities in the different components of the food system, it is recognized that it may be limited in the extent to which it can appropriately capture the exceptional elements and characteristics of Indigenous food security and food systems. The Council of Canadian Academies developed a food security and sovereignty conceptual framework that reflects the situation of Indigenous communities in Canada’s North, in the context of climate change [14]. 

## 5. Discussion

Greater understanding of the current impacts climate change is having on food security and on health is needed along with projections of future risks to populations [4]. Much of the current climate change and food security literature focuses on low-income countries [6,35]. Indeed, low-income countries will be disproportionately impacted by climate change effects, and will likely experience more severe impacts to their domestic food systems, resulting in the exacerbation of existing challenges of food insecurity and undernutrition. While high income countries like Canada generally have robust food systems, with threats such as insufficient food supply of minor concern, challenges, and stressors related to climate change impacts still exist, particularly for certain populations and regions [4,6]. 

Despite the consensus that climate change will have significant implications for global food security, research to date has primarily concentrated on one aspect of food security: availability, with a heavy focus on agricultural food production [58]. As a result, there is limited understanding of the measurable impacts climate change will have on non-production components of the food system [8]. Thus, many important aspects of food security are being overlooked both in domestic and international climate change and health research, demonstrating the need for a comprehensive conceptual framework. 

While much of the existing Canadian research on climate change impacts on food security and health is focused on communities north of 60 degrees latitude, knowledge gaps about impacts in this region continue to exist. Furthermore, very little is known about the impacts and implications for regions south of 60 degrees latitude, for Indigenous, rural, and remote communities, as well as urban centers. Given these knowledge gaps, information about health outcomes that might result from climate change impacts, and effective adaptations in the food system, is limited.

The framework demonstrates that the climate change, food security, and health nexus represent an interdisciplinary problem that requires cross-sectoral collaboration for development of effective adaptations—for example, stakeholders from transportation, agriculture, water, health, environmental conservation, natural resources, urban planning, trade, social programs, and policy and regulations all play a role in the food system. Food systems have many interdependent subsystems that operate over different time and spatial scales [22]. The disruption of one activity can impact other activities in the system. The majority of existing research ignores the interdependency of the food system [58], typically exploring subsectors of the food system in isolation from each other. The framework supports interdisciplinary and systems research, and collaboration by conceptualizing key aspects of interdependencies in the food system. 

Increasingly, the public health sector is using climate change and health vulnerability and adaptation assessments (V&As) to collect information on health risks associated with climate change and develop adaptation and response strategies [63]. The possible impacts of climate change on food security have not been widely explored in V&As within or outside of Canada. One study in 2018 that examined 34 assessments, supported by the WHO since 2013, found that all countries included an examination of climate change impacts on vector-borne diseases, while 22 countries examined water-borne diseases and only 15 investigated impacts on malnutrition [64]. 

In Canada, the 1998 assessment of health impacts of climate change did not explore the effects of climate change on food security, nor did an assessment in 2004 [65], but the 1998 study did recognize this as a high priority knowledge gap [66]. Subsequent national climate change and health assessments included examination of climate change impacts on food safety with some discussion of food insecurity threats in northern Canadian communities [4,5]. 

On the local and regional levels, risks to food security have been identified in few V&As [54,67]. In 2018, a high-level vulnerability assessment of Toronto’s food system was conducted which explored the impacts of three hypothetical extreme weather events. The assessment highlighted that extreme weather events could impact food accessibility, distribution, and food safety [68]. The report recommended developing neighborhood-level food system resiliency plans for urban centers to address the unique needs of neighborhoods that may experience increased vulnerability during extreme weather events. The findings of the assessment support the argument for a coordinated effort across the food system to address vulnerabilities. 

A food system resiliency assessment for the city of Baltimore also identified food system vulnerabilities to climate change-related hazards, and the results of the report will inform the development of a Food System Resilience Plan for the city [43]. There has yet to be a comprehensive examination of climate change impacts on Canada’s food system at the national or local levels. The proposed framework is scalable and could guide such studies, ensuring unique characteristics of Canada are considered and key vulnerabilities are not overlooked.

Adaptation can be effective in mitigating the impacts of climate change on health [69], and proven interventions exist that can enhance food security, for example, by improving food safety and by preventing or treating malnutrition [35]. However, actions taken by public health officials to prepare for climate change impacts through for example, food access or food safety programs, will have limited effectiveness in reducing poor health outcomes if other major drivers of food insecurity are not addressed. This could include appropriate measures by officials in other sectors, for example, increasing the resilience of food production facilities and transportation systems.

At the global level an adaptation gap exists in efforts to address impacts of climate change on food insecurity and nutrition. Further efforts to build the evidence base and to develop models that can assist in quantifying the burden of disease related to malnutrition associated with weather extremes are needed to ramp up efforts to protect health [35]. Watts et al. [9] suggest that human interventions play the predominant role in shaping the relationship between climate change, food insecurity, and health impacts. The relationship of people to infrastructures and markets, for example, is a greater driver of vulnerability to food insecurity than is exposure to climate-related hazards such as droughts and floods [9]. Adaptation is therefore essential to prepare for the impacts of climate change. 

The proposed framework highlights a number of important avenues of investigation related to key climate drivers, factors mediated by human behavior that influence accessibility, utilization, and availability of food and other determinants of poor health outcomes. This can aid researchers in future studies of climate change impacts on food security and health, including the development of health models and of early monitoring and warning systems of climate impacts on the food system. Investigation of unique characteristics and differences in social, political, economic, and environmental factors among urban, rural, coastal, northern, and Indigenous communities can elucidate important vulnerabilities in food systems that can inform the development of effective adaptations by health sector decision makers and those in related sectors. 

Consideration of social equity is also important when developing and implementing adaptation actions. For example, Inuit participants of a workshop that focused on developing a strategic plan for food security for the Inuvialuit Settlement Region, Northwest Territories, using the results of the 2007–2008 Inuit Health Survey, identified housing as a major concern and barrier to achieving food security [70]. In order to store and prepare healthy foods, access to affordable, adequate, and safe housing was considered critical by the participants. Housing was not initially identified by the researchers as an area of concern as it was not recognized in the health survey results [70]. Thus, signaling the importance of studying food security and human health using an interdisciplinary, comprehensive framework. Furthermore, engaging people most affected empowers them to contribute to robust responses and adaptation strategies. Future research on the climate change, food security, and human health nexus, should therefore include considerations of social equity as well as Indigenous knowledge, which contribute to the development of effective responses and adaptation action. 

## 6. Conclusions

The impacts of climate change on the Canadian food system work through complex pathways to influence human health. There is a significant need for a conceptual framework that fully captures these relationships so that Canadians and their communities can prepare for future impacts. 

The framework presented in this paper provides a comprehensive conceptualization of the climate change, food security, and human health nexus, illustrating pathways of vulnerabilities and identifying opportunities for intervention. Analyzing this nexus from a food system lens allows for a broad understanding of food security, capturing the critical dimensions and highlighting vulnerabilities that may be overlooked in the existing literature. The framework will be useful in guiding future research, and can aid in the completion of climate change and health vulnerability and adaptation assessments. It can also inform the development and implementation of adaptation and resiliency building actions.

As highlighted in the framework, the food system is an interdependent system that requires activities from numerous sectors in order to operate effectively. Thus, a collaborative, multi-sectoral response is critical in order to increase climate change resilience in the food system.

## Figures and Tables

**Table 1:** Health and social challenges associated with food insecure households, adapted from Li et al. [29].

Health Outcome Category	Health and Social Challenges Related to Food Insecurity
Birth Outcomes and Maternal Health	Negative health impacts on both the mother and baby due to inadequate nutrition during pregnancy Increased risk of birth defects Impacts on infant feeding behaviors and sustainability of breastfeeding
Child Development	Poorer general health among food insecure children Impacts on growth and development in early life Poorer academic outcomes and social skills compared to children who do not experience food insecurity Iron deficiency anemia linked to the subsequent development of a variety of chronic conditions, including asthma and depression
Health Status and Chronic Disease	Impacts on the quality and quantity of women’s food intake due to lower incomes Higher levels of poor or fair self-rated health, diabetes, heart disease, high blood pressure, food allergies and mental health outcomes Barriers to chronic disease management, increasing the likelihood of adverse outcomes
Mental Health and Emotional Well-being	Impacts on social and mental well-being that can increase the likelihood of depression, distress and social isolation Child hunger leading to depression and suicidal symptoms in adolescence and early adulthood
Economic costs	Increased health care costs associated with food insecurity In Ontario, the annual healthcare costs were 23% higher for adults in marginally food insecure households, 49% higher for those in moderately food insecure households and 121% higher for those in severely food insecure households ^1^ [34] Increased probability that adults will become high-cost users of health care

^1^ The study used data collected from 67,033 individuals aged 18–64 years in Ontario who participated in the Canadian Community Health Survey in 2005, 2007/08, and 2009/10 to assess their household food insecurity status in the 12 months before the survey interview. The authors linked this with administrative health care data to determine direct health care costs for individuals during the same 12-month period [34].

**Table 2:** Potential vulnerabilities to key food system components from climate change impacts.

Food System Component	Climate Vulnerability
Production	Increasing temperature extremes and variability, and changes in precipitation patterns and extreme weather events can cause damage to agricultural crops, reduce productivity, and decrease yield [37,38,39,47,48]
	In the Prairies, groundwater quality and quantity may be reduced as summer rainfall decreases, which can increase irrigation needs for agricultural crops [49]
	Water resources will face increasing demand and competition from other users (i.e., oil and gas industry) [49] as well as domestic demand [50], causing further impacts on water availability
	Sea level rise could cause inundation of agricultural lands in coastal regions, damaging crops, and creating unsuitable conditions for agricultural production and result in salt water intrusion of aquifers, reducing the quality of irrigation water [38]
	Increasing temperatures and changes in precipitation patterns may create more favorable conditions for pests, invasive species and plant diseases [36,37,48]
	Rising temperatures and increased concentrations of atmospheric CO_2_ may decrease the effectiveness of some herbicides used for pest control [8]
	Increasing ozone pollution, a by-product of fossil fuel combustion, can inhibit photosynthesis in plants, reducing the quality and productivity of the crop [37,48]
	Temperature extremes can adversely impact livestock health and decrease productivity [48]
	Extreme weather events may reduce land available for livestock pasture and foraging [36]
	The distribution and productivity of natural and farmed fish will change as ocean and freshwater temperatures and ocean acidification increase [8,38]
	Rising temperatures may create favorable conditions for aquatic disease and invasive species [51]
	Increasing temperatures and changing precipitation patterns are changing the quality and distribution of populations of traditionally harvested species in Canada (i.e., Caribou) [14]
Processing	Increasing temperatures and extreme temperature events may increase the risk of food spoilage or contamination while in storage at processing facilities [40]
	Traditional food storage and preparation practices may be at risk, for example, permafrost melt may have implications for the stability and safety of traditional in-ground freezers used by Indigenous communities in the Canadian North [14]
	Reduced or variable water availability may challenge food processing operations, which require significant amounts of potable water [38]
	The physical infrastructure of processing facilities may be damaged by extreme weather events (i.e., flooding) which can disrupt operations [40]
	Extreme weather events may cause disruption to energy supplies, labor availability and technological infrastructure critical to processing operations [40]
	Availability, quality, and the cost of raw materials and inputs may be variable as a result of climate change impacts in the food production sector, from both international and domestic sources [7,41]
Distribution	Extreme weather events can cause damage and disruption to transportation infrastructure including road, rail, marine, and air transport infrastructure as well as urban and public transportation systems [42]
	Temperature extremes, permafrost melt, changes in precipitation patterns, and freeze-thaw cycles can compromise the integrity of road, rail, marine, and air transport infrastructure [42]
	Extreme weather events can cause physical damage to distribution facilities (i.e., grocery stores and food banks) and also disrupt energy supplies, labor availability, and technological infrastructure critical for distribution site operations [40,41]
Preparation and Consumption	Increasing temperatures, changes in precipitation patterns, and extreme weather events can create favorable conditions for the transmission, survival, and growth of many common foodborne pathogens [40,52,53]
	Increasing temperatures may result in a rise of activities where food preparation and consumption occurs outdoors (i.e., barbeques, picnics), potentially increasing the risk of exposure to foodborne illness [40,54]
	Extreme weather events may facilitate chemical contamination at food production sites (i.e., contaminated flood waters inundating agricultural crops) [40]
	An increase in ocean temperature and changes in salinity may increase the risk of pathogens that are known to contaminate seafood (i.e., *Vibrio*) [40,52,55]
	In the Canadian North, climate change may allow for the emergence of new pathogens, viruses, and parasites that affect wildlife harvested as part of traditional and country food systems [14]
	A changing climate may increase the need for the use of pesticides which can lead to increased pesticide residues in the food supply [6]
	Increasing concentrations of atmospheric CO_2_ can alter the nutritional content of some agricultural crops, with studies indicating decreased concentration of protein, iron, zinc, and key minerals [8,40,56,57]

**Table 3:** Primary elements of food security and their critical dimensions [19].

Primary Elements of Food Security	Critical Dimensions
Food availability	Production: how much and which types of food are available
	Distribution: how food is made available (physically moved), in what form, when, and to whom
	Exchange: how much of the available food is obtained through exchange mechanisms such as barter, trade, purchases, or loans
Food accessibility	Affordability: the purchasing power of households or communities relative to the price of food
	Allocation: the economic, social, and political mechanisms governing when, where, and how food can be accessed by consumers
	Preference: social, religious, or cultural norms and values that influence consumer demand for certain types of food
Food utilization	Nutritional value: how much of the daily requirements of calories, vitamins, protein, and micronutrients are provided by the food people consume
	Social value: the social, religious, and cultural functions and benefits food provides
	Food safety: toxic contamination introduced during producing, processing, and packaging, distribution or marketing food; and foodborne diseases
